# Dynamic Network Characteristics of Power-electronics-based Power Systems

**DOI:** 10.1038/s41598-020-66635-0

**Published:** 2020-06-19

**Authors:** Yuxi Ji, Wei He, Shijie Cheng, Jürgen Kurths, Meng Zhan

**Affiliations:** 1 The State Key Laboratory of Advanced Electromagnetic Engineering and Technology, School of Electrical and Electronic Engineering, Huazhong University of Science and Technology, Wuhan, 430074 China; 2 Potsdam Institute for Climate Impact Research, Telegraphenberg, Potsdam, D-14415 Germany; 3 Institute of Physics, Humboldt University Berlin, Berlin, D-12489 Germany; 4 Saratov State University, Saratov, 4410012 Russia

**Keywords:** Engineering, Electrical and electronic engineering

## Abstract

Power flow studies in traditional power systems aim to uncover the stationary relationship between voltage amplitude and phase and active and reactive powers; they are important for both stationary and dynamic power system analysis. With the increasing penetration of large-scale power electronics devices including renewable generations interfaced with converters, the power systems become gradually power-electronics-dominant and correspondingly their dynamical behavior changes substantially. Due to the fast dynamics of converters, such as AC current controller, the quasi-stationary state approximation, which has been widely used in power systems, is no longer appropriate and should be reexamined. In this paper, for a better description of network characteristics, we develop a novel concept of dynamic power flow and uncover an explicit dynamic relation between the instantaneous powers and the voltage vectors. This mathematical relation has been well verified by simulations on transient analysis of a small power-electronics-based power system, and a small-signal frequency-domain stability analysis of a voltage source converter connected to an infinitely strong bus. These results demonstrate the applicability of the proposed method and shed an improved light on our understanding of power-electronics-dominant power systems, whose dynamical nature remains obscure.

## Introduction

Power systems have been gradually changing in its primary equipment recently with the increasing penetration of large-scale power electronics devices, including renewable energy devices such as wind turbines and photovoltaic cells, and high voltage direct current. Such a change with high shares of power electronic converters has been generally believed as the second revolution of power systems since the first established over 100 years ago, and the new-generation power system has been called power-electronics-based or power-electronics-dominant^1^. Due to the broad substitution of the primary equipment with power electronics devices, the system dynamical behavior changes substantially accompanying with the change of device characteristics. So far, a variety of serious accidents in the power-electronics-dominant power system have been happening worldwide, and their physical mechanism remains under-explored^2^. It is a great challenge to establish a new framework for the new-generation power system dynamics, and to deal with these frequently occurring oscillatory accidents. Due to the faster dynamics of controllers of power electronic converters, it becomes necessary to study the corresponding network dynamics.

The traditional power system consists of generating equipment, network, and loads, and the basic function in the power-electronics-dominant power system for energy conversion and transmission is unchanged. Both device and network characteristics are of key importance in determining the system dynamics^3–5^ For the synchronous generator (SG), which is dominant in the traditional power system, the rotor’s rotation frequency only slightly fluctuates around the working frequency due to the large inertia of the rotor. Hence, this device characteristic of SG makes it reasonable to neglect the influence of frequency variations on network parameters and treat the system variables as stationary working always around the working frequency. As a result, the classical power flow (or called load flow) calculation for uncovering the stationary relation between the amplitude and phase of the voltage and the active and reactive powers has been directly borrowed. The so-called quasi-stationary state (QSS) approximation has been broadly and successfully used in the traditional power system dynamics study, which simplifies the dynamical behavior analysis of the grid. With the so-called differential-algebraic description including differential equations for the dynamic device and algebraic equations for the stationary network, the physical picture for the traditional power system electromechanical transient becomes clear and the system complexity in analysis and calculation is greatly reduced. For some recent studies in nonlinear dynamics community under such a differential-algebraic description, such as cascading failure for power blackout and synchronization in coupled 2-order Kuromoto oscillators, see, e.g^6–16^.,

On the contrary, in power-electronics-dominant power systems, both device and network characteristics fundamentally change. In analyzing power system stability, we care more about the dynamics of equipment components and control loops in system level. The dynamics of switching components (above several kHz) is usually neglected, and thus the extremely fast switching dynamics of converters are completely not considered in this paper. In this respect, the time-scales are decomposed according to the statuses of the different equipment components or the response speeds of the different control loops after a disturbance. For instance, a direct-driven wind turbine connected to a grid through a voltage source converter (VSC) is typically featured with a multi-time scale character, namely, the AC current controller typically has the shortest time constant (around 10 ms), the DC voltage controller has a medium time constant (around 100 ms), and the mechanical speed controller has the longest time constant (around 1000 ms), depending on the level of energy storage with which the corresponding controller is associated^17^. Thus, within the fastest time scale of power-electronics devices, such as that of the AC current controller, the assumption of a stationary network may not be satisfied, since power converters, in contrast to synchronous generators, have fast time constants that are in a similar range as the time constants of electromagnetic transients. Under this situation, the dynamics of the non-reduced network should be carefully considered in the whole system analysis. Note that some studies on the propagation of disturbances in renewable energy grids still used the stationary assumption for networks^18^.

Indeed, there are several other recent studies on modeling of some detailed inductor and capacitor in networks directly by their differential and integral equations, instead of setting a constant reactance at the working frequency. Most of them focused on a small-signal stability analysis. For example, the current of each transmission line was chosen as state variables, and the system state matrix was derived in a common rotating DQ frame by combining the state equation of the network with other components such as converters and loads^19,20^ The network dynamics was considered under the relation between the node current and voltage, and the admittance matrix of the network in the frequency domain was obtained^21^. In^22^, the small-signal frequency domain model of network in the polar coordinates was established, based on a transfer function matrix defined by a generalized impedance concept. A few models of dynamic network have also been built and studied numerically in electromagnetic transient simulations^23,24^ In addition, the concept of dynamic phasor was proposed to generalize the idea of quasi-static phasors, by representing voltage and current signals by Fourier series expansions in which the harmonic components are evaluated over a moving time window^25^. This gives an accurate representation of the system, while using a relatively larger numerical step size compared to the electromagnetic transient simulations^26^. In^27^, power networks were modeled using dynamic phasors in the dq0 reference frame, which offers a high accuracy and fast simulation. It is notable that most of these studies relied on the direct relation between current and voltage, and focused on the small-signal stability. However, we would like to emphasize that the essence of oscillations in a power system is power conversion. A direct relation between current and voltage may fail to reflect the contribution of networks as an energy transfer element to the system oscillations. In order to have a better understanding of the oscillation mechanism in power-electronics-dominant power systems, we need an extension from the stationary power flow. Note that along this line, Zhang *et al*. have made a fundamental contribution by proposing a power-synchronization control and a Jacobian transfer matrix approach for the network dynamics, but still within the framework of small-signal stability^28–30^.

**Figure 1:**
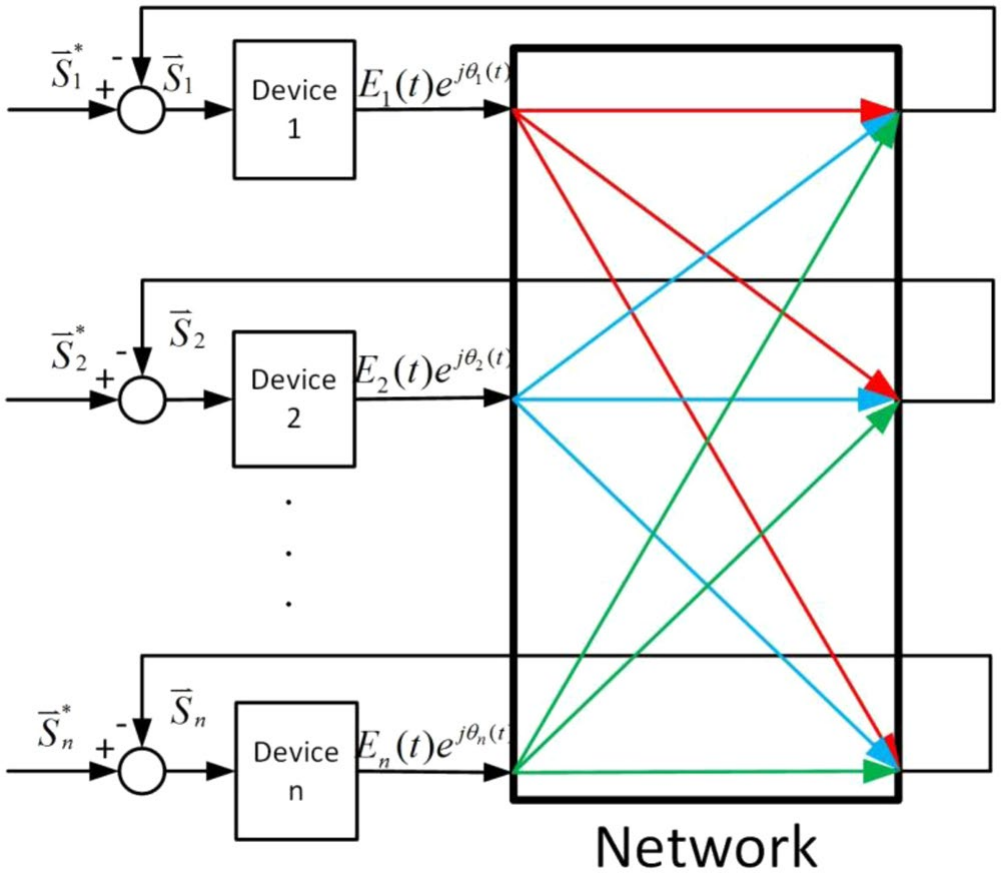
Simplified framework for a functionally connected power system, including various types of devices and network. In the whole system, each device acts as a rotating electromotive force, ${E}_{{\rm{i}}}(t){e}^{j{\theta }_{i}(t)}$, and its dynamic behavior should be determined by the imbalanced complex powers, ${\bar{{\rm{S}}}}_{i}$, including both active and reactive powers, and correspondingly the network acts as a reservoir for the interaction of each device states by transforming their electromagnetic powers. For a dynamic analysis in the traditional power systems, generally the network interaction is described by stationary power flow, whereas for power-electronics-dominant power systems, the dynamic power flow induced by the fast time-scale behavior of the devices must be considered. For more details, see the text.

In the studies of power-electronics-dominant power system dynamics, a novel method of so-called amplitude-phase motion equation has been proposed and developed very recently^31–35^ Basically, it borrows the electromechanical motion equation for the SG’s rotor, the swing equation, namely, the rotor’s motion (or state) is solely determined by the imbalance between the input mechanical power and the electromagnetic power, and intends to describe the dynamics (motion equation) of various types of power electronics devices in a unified mechanical-like way. From a system science point of view, each device should be expressed by its unique external characteristics as an electromotive force (EMF). Correspondingly, the network, as usual, works as a reservoir for the interaction of all these devices connected to the grid. To sum up, the amplitude-phase motion equation method views each device by taking the imbalanced powers as input, and the amplitude and phase of the EMF as output, and meanwhile it treats the network by taking EMF as input and power as output. A schematic showing the interaction of devices through the network is illustrated in Fig. 1. It establishes a functional connection between various power-electronics devices in a general, unified manner, and focuses on the external characteristics of devices and network by simple input-output relations. Until now in this direction, several models of devices, such as wind turbine at different time scales, have been well established^31,34,35^ To further study the whole system dynamics as a closed loop under the same framework, we need to develop a dynamic network model, which is emphasized by the large black box in Fig. 1.

In the paper, we will concentrate on the dynamic network characteristics of power systems. The resistance of typical overhead transmission line is usually around one tenth of its reactance. We assume that the network is purely inductive for simplicity, since inductors are the main parts of transmission lines in transmission networks working on several hundred kV^3^, and the results can be easily extended to include resistive elements of transmission lines. Similar to the power flow concept in traditional power systems, it characterizes the relation between the active and reactive powers and the amplitude and phase of the bus voltage. Nevertheless, differently now all voltage states have a time-varying frequency (not constant fundamental frequency) and should be treated as a space vector (not phasor, which is valid and built on the fundamental frequency). The restriction for a sufficiently slow frequency variation used in the traditional power system could be removed. The so-obtained dynamical relation between the power and voltage vector is expected to be general and appropriate not only for traditional power systems, but also for power-electronics-dominant ones. The essence of the dynamic process of power systems is the interaction of imbalanced powers and system states. Describing the characteristic of devices and networks in the model of amplitude-angle motion equation reflects their own contribution in such a process. As a result, the complicated dynamic phenomena and mechanism in power systems are expected to be clear in a physical and unified manner.

## Results

### Stationary power flow in traditional power systems

In the conventional power system analysis, the power flow study involves the calculation of power flows and voltages of a transmission network for specified terminal or bus conditions, and it is fundamental for a steady-state as well as a dynamic performance of power systems^5,3,4^

Considering that the instantaneous frequency *ω*_*i*_ of node voltage

1 $${\omega }_{i}(t)=\frac{d{\theta }_{i}(t)}{dt}$$

always fluctuates around the working frequency *ω*_0_ (*ω*_0_ = 1*00π* rad/s), i.e.,

2 $${\theta }_{i}(t)={\omega }_{0}t+{\delta }_{i}(t),$$

where *δ*_*i*_*(t)* usually refers to the node voltage angle. We can treat all system variables as phasors. Denote **E**_**i**_ as the phasor voltage to ground at node *i*, and **I**_**i**_ as the phasor current flowing into the network at node *i*, we have the expression for the complex power

3 $${\bar{{\rm{S}}}}_{i}={P}_{i}+j{Q}_{i}={E}_{i}{I}_{i}^{\ast }$$

and

4 $${{\rm{I}}}_{i}=\mathop{\sum }\limits_{j=1}^{n}{Y}_{ij}{E}_{j}=\mathop{\sum }\limits_{j=1}^{n}({G}_{ij}+j{B}_{ij}){E}_{j}$$

where *Y*_*ij*_ represents mutual admittance between nodes *i* and *j* (*Y*_*ii*_ for self-admittance of node *i*), and *G*_*ij*_ and *B*_*ij*_ are its conductance and susceptance, respectively.

Hence, we can get the well-known power-flow equations

5 $$\{\begin{array}{c}{P}_{i}={E}_{i}\mathop{\sum }\limits_{j=1}^{n}({G}_{ij}{E}_{j}\,\cos ({\delta }_{i}-{\delta }_{j})+{B}_{ij}{E}_{j}\,\sin ({\delta }_{i}-{\delta }_{j}))\\ {Q}_{i}={E}_{i}\mathop{\sum }\limits_{j=1}^{n}({G}_{ij}{E}_{j}\,\sin ({\delta }_{i}-{\delta }_{j})-{B}_{ij}{E}_{j}\,\cos ({\delta }_{i}-{\delta }_{j}))\end{array}$$

and their simplified form,

6 $$\{\begin{array}{c}{P}_{i}={E}_{i}\mathop{\sum }\limits_{j=1}^{n}\frac{{E}_{j}}{{X}_{ij}}\,\sin ({\delta }_{i}-{\delta }_{j})\\ {Q}_{i}={E}_{i}\mathop{\sum }\limits_{j=1}^{n}\frac{{E}_{j}}{{X}_{ij}}\,\cos ({\delta }_{i}-{\delta }_{j})\end{array}$$

if the situation of very high-voltage loss-less transmission network is considered, based on

$${G}_{ij}=0,\,{B}_{ij}=-\,1/{X}_{ij}$$

where *X*_*ij*_ represents reactance between nodes *i* and *j*.

Numerically solving the above stationary power flow is basic and important for us to know the power distribution and the bus voltage information^3–5^. The classical Gauss-Seidel, Newton-Raphson, and P-Q decoupled algorithms have been applied to deal with these nonlinear algebraic equations^3^. Importantly the power flow calculation has also been generalized to study economic dispatch problem (or the minimum-loss problem) by means of optimal power flow (OPF)^36^, and the steady-state voltage stability problem within the continuation power flow (CPF)^37^. Thus, it plays a central role in the traditional power system analysis.

In addition, in the study of the dynamic performance of power systems, including the small-signal stability and transient stability, the stationary power flow calculation has also been directly used. For instance, for the simplest power system with a SG whose internal potential amplitude is *E* and its phase is *δ*_*1*_, connected to an infinitely strong bus, whose bus voltage amplitude is fixed as *V* and its voltage phase is fixed at zero (*δ*_2_ = 0), we have

7 $$\{\begin{array}{c}P=\frac{EV}{X}\,\sin ({\delta }_{1}-{\delta }_{2})\\ Q=\frac{{E}^{2}}{X}-\frac{EV}{X}\,\cos ({\delta }_{1}-{\delta }_{2})\end{array}$$

Usually the first equation for the sinusoidal relation between the phase angle and active power is well-known, called phase-angle relation (or phase-angle curve)^3–5^ Combined with the SG’s swing equation, it has been used to describe the basic physical picture of small-signal stability, such as the system is stable under *δ* < *π/2* and otherwise it is unstable, and that of transient stability based on the equal-area criterion as well. Except these, the power flow has also been directly used even in the low-frequency oscillation analysis, based on the fact that the oscillation frequency is much lower than the working frequency. In addition, the second equation for the relation between the voltage magnitude and the reactive power has been used in the (stationary) voltage stability to provide a basic physical picture. As a result, the power flow is fundamental and invaluable for our understanding of power system dynamics.

### Dynamic power flow in power-electronics- dominant power systems

**Figure 2:**
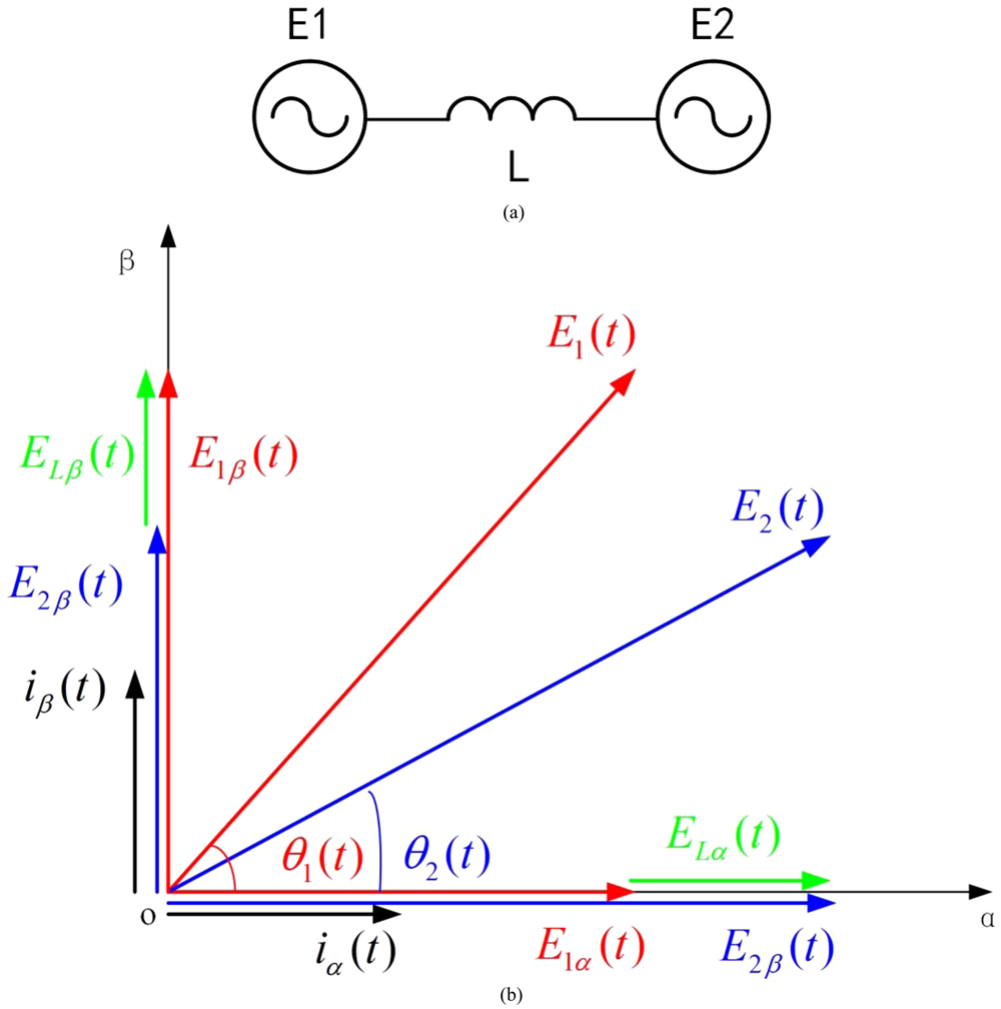
(**a**) A small power system consisting of two time-varying voltage sources, connected by a transmission line represented by a constant inductance *L*. (**b**) Schematic show for the instantaneous vector relation between voltages and current.

In this section, we will go a step beyond and study the dynamic network characteristics, as the electromagnetic time scale of power-electronics devices is much faster than the electromechanical time scale, such as the rotor motion of the SG. The resistance of typical overhead transmission line is usually around one tenth of its reactance^3^. Thus, to simplify the study, we assume that the transmission line is working on high voltage by ignoring its resistance and capacitance. This assumption is suitable for transmission lines in high-voltage transmission networks. First, we derive the dynamic power flow relation in the time domain, by starting from a small system consisting of two voltage sources connected with a transmission line shown in Fig. 2(a), where **E**_**1**_ and **E**_**2**_ stand for the two time-varying voltage vectors. Then the corresponding simple relation is generalized to multi-machine systems based on the superposition theory of linear systems. Finally, the time domain relation is linearized to describe small-signal stability in the frequency domain, working as a transfer function matrix of the network.

As we know, in this case the usual QSS approximation for a constant frequency for all bus voltage phasors is non-workable, and we have to deal with the interaction of time-varying voltage vectors directly. Meanwhile, the stationary relation between voltage and current connected by a reactance on the working frequency should be replaced by a direct dynamic relation between voltage and current on inductance, whose value is a constant *L*. Comparatively, the dynamical relation is more essential. Correspondingly, the usual average power concepts including active and reactive powers should also be generalized to instantaneous power concepts^38^. With the help of the instantaneous power theory and an addition of auxiliary variables, we obtain the instantaneous dynamic relation between powers and voltage vectors.

## Nonlinear Relation in the Time Domain

In the simple system in Fig. 2(a), we should first examine the current dynamics based on the voltage vectors on a transmission line, with a constant inductance *L*. It is the same as we have done in the stationary power flow analysis. It can be expressed in the α-β stationary frame

8 $$\{\begin{array}{c}{i}_{\alpha }(t)=\frac{1}{L}\int [{E}_{1\alpha }(t)-{E}_{2\alpha }(t)]dt=\frac{1}{L}\int [{E}_{1}(t)\cos \,{\theta }_{1}(t)-{E}_{2}(t)\cos \,{\theta }_{2}(t)]dt\\ {i}_{\beta }(t)=\frac{1}{L}\int [{E}_{1\beta }(t)-{E}_{2\beta }(t)]dt=\frac{1}{L}\int [{E}_{1}(t)\sin \,{\theta }_{1}(t)-{E}_{2}(t)\sin \,{\theta }_{2}(t)]dt\end{array}$$

A schematic show is given in Fig. 2(b) for the instantaneous vector relation between voltages and current. The red lines stand for the time-varying vector *E*_1_ and its instantaneous values in the stationary frame, and the blue lines stand for that of *E*_2_. The green lines refer to the voltage difference *E*_*L*_ on *L* in the stationary frame, and the current *i*_*α*_ and *i*_*β*_ can be obtained by the integral of *E*_*Lα*_ and *E*_*Lβ*_ (the black lines). It is important to note that both the voltage amplitude *E*_*1,2*_ and voltage phase *θ*_*1,2*_ are time-varying, and here we treat an arbitrary phase *θ*(*t*) (not the phasor phase *δ*(*t*) in the stationary power flow).

Since the voltage and the current are not necessarily periodic, the concept of average power is no longer available. By using the instantaneous power theory^38^, we further get

9 $$\{\begin{array}{c}{P}_{1}(t)={E}_{1}(t)I(t)\cos \,[{\theta }_{1}(t)-{\theta }_{i}(t)]={E}_{1\alpha }(t){i}_{\alpha }(t)+{E}_{1\beta }(t){i}_{\beta }(t)\\ {Q}_{1}(t)={E}_{1}(t)I(t)\sin \,[{\theta }_{1}(t)-{\theta }_{i}(t)]={E}_{1\beta }(t){i}_{\alpha }(t)-{E}_{1\alpha }(t){i}_{\beta }(t)\end{array}$$

which, after substituting(8) into (9), yields

10 $$\{\begin{array}{ccc}{P}_{1}(t) & = & \frac{{E}_{1}(t)}{L}\{\cos \,{\theta }_{1}(t)\int {E}_{1}(t)\cos \,{\theta }_{1}(t)dt+\,\sin \,{\theta }_{1}(t)\int {E}_{1}(t)\sin \,{\theta }_{1}(t)dt\\  &  & -[\,\cos \,{\theta }_{1}(t)\int {E}_{2}(t)\cos \,{\theta }_{2}(t)dt\,+\,\sin \,{\theta }_{1}(t)\int {E}_{2}(t)\sin \,{\theta }_{2}(t)dt]\}\\ {Q}_{1}(t) & = & \frac{{E}_{1}(t)}{L}\{\sin \,{\theta }_{1}(t)\int {E}_{1}(t)\cos \,{\theta }_{1}(t)dt-\,\cos \,{\theta }_{1}(t)\int {E}_{1}(t)\sin \,{\theta }_{1}(t)dt\\  &  & -[\,\sin \,{\theta }_{1}(t)\int {E}_{2}(t)\cos \,{\theta }_{2}(t)dt\,-\,\cos \,{\theta }_{1}(t)\int {E}_{2}(t)\sin \,{\theta }_{2}(t)dt]\}\end{array}$$

These integration relations are very complex and hard to analyze. In the sub-field of mathematics, harmonic analysis, such an integral is a so-called oscillatory integral, whose primary function cannot be analytically derived^39^. To solve this difficulty, we introduce an auxiliary complex variable, *z*, with *m*(*t*) and *n*(*t*) as their real and imaginary parts, respectively, and have the relations between *z* (*t*), *m*(*t*), and *n*(*t)*

11 $$z=m(t)+n(t)j={e}^{j{\theta }_{1}(t)}\int ({E}_{1}(t){e}^{-j{\theta }_{1}(t)}-{E}_{2}(t){e}^{-j{\theta }_{2}(t)})dt$$

and further the relation between m(t) (and n(t)) and the voltage vector (E(t) and θ(t))

12 $$\{\begin{array}{c}m(t)=\,\cos \,{\theta }_{1}(t)\int ({E}_{1}(t)\cos \,{\theta }_{1}(t)-{E}_{2}(t)\cos \,{\theta }_{2}(t))dt+\sin \,{\theta }_{1}(t)\int ({E}_{1}(t)\sin \,{\theta }_{1}(t)-{E}_{2}(t)\sin \,{\theta }_{2}(t))dt\\ n(t)=\,\sin \,{\theta }_{1}(t)\int ({E}_{1}(t)\cos \,{\theta }_{1}(t)-{E}_{2}(t)\cos \,{\theta }_{2}(t))dt-\,\cos \,{\theta }_{1}(t)\int ({E}_{1}(t)\sin \,{\theta }_{1}(t)-{E}_{2}(t)\sin \,{\theta }_{2}(t))dt\end{array}$$

Taking a derivative of *z*(*t*), we obtain

13 $$\frac{dz}{dt}=j{\omega }_{1}(t)z(t)+{E}_{1}(t)-{E}_{2}(t){e}^{j({\theta }_{1}(t)-{\theta }_{2}(t))}$$

which can be further written into the time derivative of the real and imaginary parts, *m(t)* and *n(t)*, respectively,

14 $$\{\begin{array}{c}\frac{dm}{dt}=-{\omega }_{1}(t)n(t)+{E}_{1}(t)-{E}_{2}(t)\cos \,{\theta }_{12}(t)\\ \frac{dn}{dt}={\omega }_{1}(t)m(t)-{E}_{2}(t)\sin \,{\theta }_{12}(t)\end{array}$$

where $${\theta }_{12}(t)={\theta }_{1}(t)-{\theta }_{2}(t)$$.

Therefore, the complicated integration relations in (10) can be expressed as the following simpler algebraic relations

15 $$\{\begin{array}{c}{P}_{1}(t)=\frac{{E}_{1}(t)}{L}m(t)\\ {Q}_{1}(t)=\frac{{E}_{1}(t)}{L}n(t)\end{array}$$

where ${P}_{1}(t)$ and ${Q}_{1}(t)$ are a function of $m(t)$ and $n(t)$, respectively, with the same time-varying coefficient ${E}_{1}(t)/L$.

The above differential algebraic equations containing two differential Eq. (14) and two algebraic Eq. (15) could fully catch the dynamic network characteristics and play the same role as the familiar stationary power flow in the traditional power system. Meanwhile, compared to the stationary power-flow algebraic relation in Eq. (7), the dynamic relation described by Eqs. (14) and (15) becomes more complicated.

Next, we study the stationary relation by setting the left side of (14) equals to zero and have

16 $$\{\begin{array}{c}{m}_{0}=\frac{{E}_{20}\,\sin ({\theta }_{10}-{\theta }_{20})}{{\omega }_{0}}\\ {n}_{0}=\frac{{E}_{10}-{E}_{20}\,\cos ({\theta }_{10}-{\theta }_{20})}{{\omega }_{0}}\end{array}$$

and further

17 $$\{\begin{array}{c}{P}_{0}=\frac{{E}_{10}}{L}{m}_{0}=\frac{{E}_{10}{E}_{20}}{{\omega }_{0}L}\,\sin ({\theta }_{10}-{\theta }_{20})\\ {Q}_{0}=\frac{{E}_{10}}{L}{n}_{0}=\frac{{E}_{10}}{{\omega }_{0}L}-\frac{{E}_{10}{E}_{20}}{{\omega }_{0}L}\,\cos ({\theta }_{10}-{\theta }_{20})\end{array}$$

which are identical to the results of the stationary power flow in (7), as *X* = *ω*_0_*L* and *θ*_1_*(t)-θ*_2_*(t)* = *δ*_1_*(t)-δ*_2_*(t)*. This indicates that the results obtained in the paper for the dynamic power flow can really be reduced to those for the stationary power flow.

After the analysis of the dynamic power flow in a small power system, we extend this result to more complicated and realistic larger power systems. By using the superposition theorem in the linear inductance circuit excited by multiply voltage sources (signals), we get the following dynamic relations for multi-machine systems

18 $$\{\begin{array}{c}{P}_{i}(t)={E}_{i}(t)\mathop{\sum }\limits_{\begin{array}{c}j=1\\ j\ne i\end{array}}^{n-1}\left(\frac{{m}_{ij}(t)}{{L}_{ij}}\right)\\ \begin{array}{c}{Q}_{i}(t)={E}_{i}(t)\mathop{\sum }\limits_{\begin{array}{c}j=1\\ j\ne i\end{array}}^{n-1}\left(\frac{{n}_{ij}(t)}{{L}_{ij}}\right)\\ \frac{d{m}_{ij}}{dt}=-\,{\omega }_{i}(t){n}_{ij}(t)+{E}_{i}(t)-{E}_{j}(t)\cos \,[{\theta }_{i}(t)-{\theta }_{j}(t)]\,(i\ne j)\\ \frac{d{n}_{ij}}{dt}={\omega }_{i}(t){m}_{ij}(t)-{E}_{j}(t)\sin \,[{\theta }_{i}(t)-{\theta }_{j}(t)]\,(i\ne j)\end{array}\end{array}$$

Note that in the above derivations we indeed consider the interaction of multiple voltage sources, which are truly time-varying signals, and have not made any additional assumption or simplification. Therefore, these time-domain equations are expected to be rigorous and applicable for a general dynamical analysis of power systems, such as large signal stability.

## Small-signal Linearized Relation in Frequecy Domain

Based on the above time-domain nonlinear relations, we can easily derive the small-signal linearized relations in the frequency-domain around the steady state. We still start from the simple power system in Fig. 2(a), replace the differential operator in the time-domain with the Laplacian operator *s*, and obtain

19 $$\{\begin{array}{rcl}\Delta {P}_{1} & = & \frac{{E}_{10}}{L}\Delta m+\frac{{m}_{0}}{L}\Delta {E}_{1}\\ \Delta {Q}_{1} & = & \frac{{E}_{10}}{L}\Delta n+\frac{{n}_{0}}{L}\Delta {E}_{1}\\ \Delta m & = & \frac{-{n}_{0}{s}^{2}-({E}_{20}\,\sin \,{\theta }_{120}+{m}_{0}{\omega }_{0})s-{\omega }_{0}{E}_{20}\,\cos \,{\theta }_{120}}{{s}^{2}+{\omega }_{0}^{2}}\Delta {\theta }_{1}+\frac{s}{{s}^{2}+{\omega }_{0}^{2}}\Delta {E}_{1}\\  &  & -\frac{\cos \,{\theta }_{120}s-{\omega }_{0}\,\sin \,{\theta }_{120}}{{s}^{2}+{\omega }_{0}^{2}}\Delta {E}_{2}-\frac{{E}_{20}\,\sin \,{\theta }_{120}s+{\omega }_{0}{E}_{20}\,\cos \,{\theta }_{120}}{{s}^{2}+{\omega }_{0}^{2}}\Delta {\theta }_{2}\\ \Delta n & = & \frac{{m}_{0}{s}^{2}-({E}_{20}\,\cos \,{\theta }_{120}+{n}_{0}{\omega }_{0})s+{\omega }_{0}{E}_{20}\,\sin \,{\theta }_{120}}{{s}^{2}+{\omega }_{0}^{2}}\Delta {\theta }_{1}+\frac{{\omega }_{0}}{{s}^{2}+{\omega }_{0}^{2}}\Delta E\\  &  & -\frac{\sin \,{\theta }_{120}s+{\omega }_{0}\,\cos \,{\theta }_{120}}{{s}^{2}+{\omega }_{0}^{2}}\Delta {E}_{2}-\frac{-{E}_{20}\,\cos \,{\theta }_{120}s+{\omega }_{0}{E}_{20}\,\sin \,{\theta }_{120}}{{s}^{2}+{\omega }_{0}^{2}}\Delta {\theta }_{2}\end{array}$$

where all the steady states have been expressed with the subscript zero, and *θ*_120_ = *θ*_10_ − *θ*_20_. None of the steady states is time varying.

Further removing the intermediate variables *Δm* and *Δn*, we get the explicit expression of the active and reactive powers *P* and *Q* as a function of the voltage amplitude *E*_*1,2*_ and voltage phase *θ*_*1,2*_ in the frequency domain as a transfer function matrix

20 $$\{\begin{array}{rcl}\Delta {P}_{1} & = & \frac{\frac{{E}_{20}\,\sin \,{\theta }_{120}}{{\omega }_{0}}{s}^{2}+{E}_{10}s+{\omega }_{0}{E}_{20}\,\sin \,{\theta }_{120}}{L({s}^{2}+{\omega }_{0}^{2})}\Delta {E}_{1}+\frac{\frac{{E}_{10}{E}_{20}\,\cos \,{\theta }_{120}-{E}_{10}^{2}}{{\omega }_{0}}{s}^{2}+{\omega }_{0}{E}_{10}{E}_{20}\,\cos \,{\theta }_{120}}{L({s}^{2}+{\omega }_{0}^{2})}\Delta {\theta }_{1}\\  &  & -\frac{{E}_{10}\,\cos \,{\theta }_{120}s-{\omega }_{0}{E}_{10}\,\sin \,{\theta }_{120}}{L({s}^{2}+{\omega }_{0}^{2})}\Delta E{}_{2}-\frac{{E}_{10}{E}_{20}\,\sin \,{\theta }_{120}s+{\omega }_{0}{E}_{10}{E}_{20}\,\cos \,{\theta }_{120}}{L({s}^{2}+{\omega }_{0}^{2})}\Delta {\theta }_{2}\\ \Delta {Q}_{1} & = & \frac{\frac{{E}_{10}-{E}_{20}\,\cos \,{\theta }_{120}}{{\omega }_{0}}{s}^{2}+{\omega }_{0}(2{E}_{10}-{E}_{20}\,\cos \,{\theta }_{120})}{L({s}^{2}+{{\omega }_{0}}^{2})}\Delta {E}_{1}+\frac{\frac{{E}_{10}{E}_{20}\,\sin \,{\theta }_{120}}{{\omega }_{0}}{s}^{2}-{E}_{10}^{2}s+{\omega }_{0}{E}_{10}{E}_{20}\,\sin \,{\theta }_{120}}{L({s}^{2}+{{\omega }_{0}}^{2})}\Delta {\theta }_{1}\\  &  & -\frac{{E}_{10}\,\sin \,{\theta }_{120}s+{\omega }_{0}{E}_{10}\,\cos \,{\theta }_{120}}{L({s}^{2}+{\omega }_{0}^{2})}\Delta {E}_{2}+\frac{{E}_{10}{E}_{20}\,\cos \,{\theta }_{120}s-{\omega }_{0}{E}_{10}{E}_{20}\,\sin \,{\theta }_{120}}{L({s}^{2}+{\omega }_{0}^{2})}\Delta {\theta }_{2}\end{array}$$

which definitely catches the dynamic network characteristics under the linearized condition. It is important to emphasize that it has the same form as that in the Jacobian transfer matrix in^28^.

We can further apply the same idea to multi-machine systems, which are connected by multiple transmission lines, and obtain

21 $$\{\begin{array}{rcl}\Delta {P}_{i} & = & (\mathop{\sum }\limits_{\begin{array}{c}j=1\\ j\ne i\end{array}}^{n-1}\frac{{E}_{j0}\,\sin \,{\theta }_{ij0}{s}^{2}+{\omega }_{0}{E}_{i0}s+{\omega }_{0}^{2}{E}_{j0}\,\sin \,{\theta }_{ij0}}{{\omega }_{0}{L}_{ij}({s}^{2}+{\omega }_{0}^{2})})\Delta {E}_{i}+(\mathop{\sum }\limits_{\begin{array}{c}j=1\\ j\ne i\end{array}}^{n-1}\frac{({E}_{i0}{E}_{j0}\,\cos \,{\theta }_{ij0}-{E}_{j0}^{2}){s}^{2}+{\omega }_{0}^{2}{E}_{i0}{E}_{j0}\,\cos \,{\theta }_{ij0}}{{\omega }_{0}{L}_{ij}({s}^{2}+{\omega }_{0}^{2})})\Delta {\theta }_{i}\\  &  & -{E}_{i0}\mathop{\sum }\limits_{\begin{array}{c}j=1\\ j\ne i\end{array}}^{n-1}(\frac{\cos \,{\theta }_{ij0}s-{\omega }_{0}\,\sin \,{\theta }_{ij0}}{({s}^{2}+{\omega }_{0}^{2}){L}_{ij}}\Delta {E}_{j})-{E}_{i0}\mathop{\sum }\limits_{\begin{array}{c}j=1\\ j\ne i\end{array}}^{n-1}(\frac{{E}_{j0}\,\sin \,{\theta }_{ij0}s+{\omega }_{0}{E}_{j0}\,\cos \,{\theta }_{ij0}}{({s}^{2}+{\omega }_{0}^{2}){L}_{ij}}\Delta {\theta }_{j})\\ \Delta {Q}_{i} & = & (\mathop{\sum }\limits_{\begin{array}{c}j=1\\ j\ne i\end{array}}^{n-1}\frac{({E}_{i0}-{E}_{j0}\,\cos \,{\theta }_{ij0}){s}^{2}+{\omega }_{0}^{2}(2{E}_{i0}-{E}_{j0}\,\cos \,{\theta }_{ij0})}{{\omega }_{0}{L}_{ij}({s}^{2}+{\omega }_{0}^{2})})\Delta {E}_{i}+(\mathop{\sum }\limits_{\begin{array}{c}j=1\\ j\ne i\end{array}}^{n-1}\frac{{E}_{i0}{E}_{j0}\,\sin \,{\theta }_{ij0}{s}^{2}-{E}_{i0}^{2}s+{\omega }_{0}^{2}{E}_{i0}{E}_{j0}\,\sin \,{\theta }_{ij0}}{{\omega }_{0}{L}_{ij}({s}^{2}+{\omega }_{0}^{2})})\Delta {\theta }_{i}\\  &  & -{E}_{i0}\mathop{\sum }\limits_{\begin{array}{c}j=1\\ j\ne i\end{array}}^{n-1}(\frac{\sin \,{\theta }_{ij0}s+{\omega }_{0}\,\cos \,{\theta }_{ij0}}{({s}^{2}+{\omega }_{0}^{2}){L}_{ij}}\Delta {E}_{j})-{E}_{i0}\mathop{\sum }\limits_{\begin{array}{c}j=1\\ j\ne i\end{array}}^{n-1}(\frac{{E}_{j0}\,\cos \,{\theta }_{ij0}s-{\omega }_{0}{E}_{j0}\,\sin \,{\theta }_{ij0}}{({s}^{2}+{\omega }_{0}^{2}){L}_{ij}}\Delta {\theta }_{j})\end{array}$$

which construct a multiple-input-multiple-output (MIMO) transfer matrix and explicitly catch the dynamic network characteristics of multi-machine power systems.

## Methods

**Figure 3:**
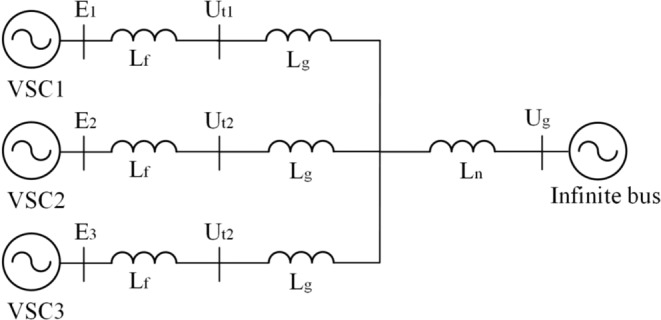
A small power-electronics-based power system consisting of three VSCs connected to an infinite strong bus.

So far, we have obtained the dynamic power flow equations for the description of dynamic network characteristics, including the original time-domain nonlinear relation and the frequency-domain linearized relation. These theoretical results need to be further verified and specified. As two typical examples presented in the paper, in the time-domain verification, we study the dynamic interaction in a real small power-electronics-based power system consisting of three VSCs connected to an infinite bus in Fig. 3. In the frequency-domain verification, we study the small-signal stability of a VSC connected to an infinitely strong bus under the AC current control time scale, whose multivariable frequency-domain analysis result will be compared with that of the state-space eigenvalue analysis. In addition, some other examples have also been examined.

### Validity test for original time-domain nonlinear relation

The system studied in Fig. 3 is a paradigm for renewable energy integration to power grids. The system consists of three VSCs connected to an infinite bus. The VSC has four control loops: DC capacitor voltage control, terminal voltage control, PLL control, and current control. For simplicity, the VSCs work at the same work point and the control parameters of them are all the same. The detailed parameters are listed in Table 1.

**Table 1:** Studied system parameters of Fig. 3 p.u. (based on 100 rad/s 2 MW and 690 V).

Symbol	Quantity	Value
*U*_g_	Grid voltage	0.8165
*P*_m_	Active power reference	0.3
*U*_t_	Teminal voltage reference	0.8165
*L*_f_	Filter inductor	0.1
*L*_g_	Network inductor	0.05
*L*_n_	Transmission line inductor	0.2
*C*	DC capacitor	0.1(F)
*U*_dc_	DC capacitor voltage	1200(V)
*K*_p1_,*K*_i1_	DC capacitor voltage power control	*K*_p1_ = 3.5,*K*_i1_ = 100
*K*_p2_,*K*_i2_	Teminal voltage control	*K*_p2_ = 1,*K*_i2_ = 100
*K*_p3_*,K*_i3_	Current control	*K*_p3_ = 0.36,*K*_i3_ = 192
*K*_p4_*,K*_i4_	PLL control	*K*_p4_ = 50,*K*_i4_ = 2000

**Figure 4:**
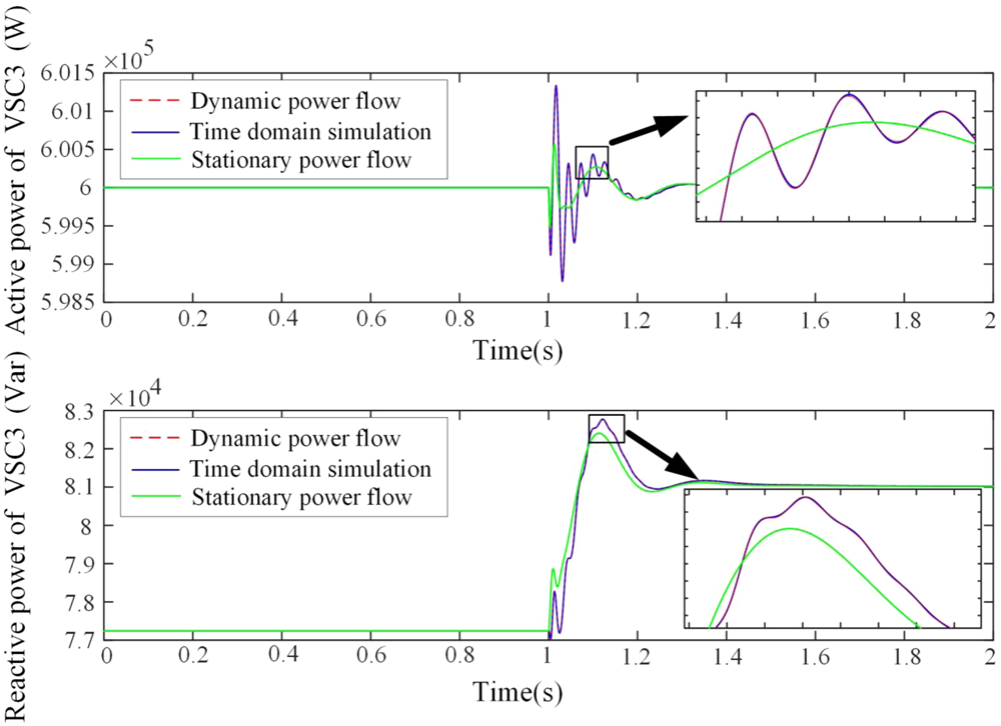
Comparison of dynamic power flow, time domain simulation, and stationary load flow, which confirms the validity of the original time-domain relation in the dynamic power flow theory.

In our test, the system keeps running in a steady state at first, and after 1 second the active power reference of the first VSC changes from 0.30 p.u. to 0.33 p.u. suddenly. Therefore, the system must be in a transient process until it enters another steady state or becomes unstable. For illustration, Fig. 4 compares the results of active and reactive powers of the third VSC, by calculating the dynamic power flow in the differential algebraic Eq. (18) by numerical integration (red dashed curve) and performing the time-domain simulation with the aid of the Simulink (blue solid curve). In addition, the result of stationary power flow (green solid curve) is also given. With these comparisons in the figures and especially in the magnification plots of the transient peaks, clearly the results based on the theory of dynamic power flow and time domain simulation coincide, whereas the result of the stationary power flow shows a large deviation. This verifies that our theory can reflect the system dynamic very well, while the traditional stationary power flow only can reflect the slow dynamics of system. Extensive numerical simulations for other cases also support this point.

### Validity test for frequency-domain linearized relation

**Figure 5:**
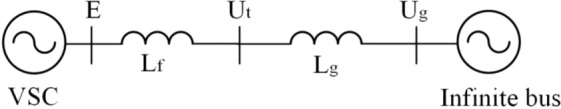
Schematic show for a simple power-electronics-dominant power system with a single VSC connected to an infinitely strong bus, where the symbols *E*, *U*_*t*_, and *U*_*g*_ represent the internal potential, terminal voltage, and voltage of the infinite bus, respectively.

As a typical model of a power-electronics-dominant power system, a single VSC connected to an infinitely strong bus is chosen for the frequency-domain verification. A schematic show for the model is given in Fig. 5. As we are particularly interested in the dynamic network characteristics, we will mainly focus on the current control time scale dynamics^35^. The typical parameters are listed in Table 2. Under this situation, we determine the transfer function matrix of the network from Eq. (20). In the framework of amplitude-phase motion equation theory, the output voltage of a device is defined as its internal voltage, since the internal voltage’s dynamic is only determined by the device itself^35^. To be consistent with the analytical results in the previous studies of the amplitude-phase model, now the inputs and outputs of the transfer matrix are the voltage vectors and powers, respectively, at the internal potential (*E*) instead of the terminal voltage (*U*_*t*_). Namely, the interaction between the VSC and the network is believed as happening at the exit point of the internal potential. Therefore, the network conductance *L* contains both *L*_*f*_ and *L*_*g*_. The transfer matrix of the network is derived by using Eq. (20), and the detailed derivation of that of the VSC is given in the appendix. For the system stability of the whole close-loop system, we investigate it with the help of the generalized Nyquist criterion on the open-loop transfer matrix of the network^40^.

**Table 2:** Studied System Parameter s of Fig. 5 p.u. (based on 100 rad/s 2 MW and 690 V).

Symbol	Quantity	Value
*U*_g_	Grid voltage	1.0
*P*	Active power reference	1.0
*U*_t_	Teminal voltage reference	1.0
*L*_f_	Filter inductor	0.1
*L*_g_	Network	Relatively Stiff Grid: 0.5 Almost Critical Condition: 0.85 Relatively Weak Grid: 1.5
*K*_p1_,*K*_i1_	Current control	*K*_p1_ = 0.3,*K*_i1_ = 160
*K*_p2_,*K*_i2_	PLL control	*K*_p2_ = 50,*K*_i2_ = 2000

**Figure 6:**
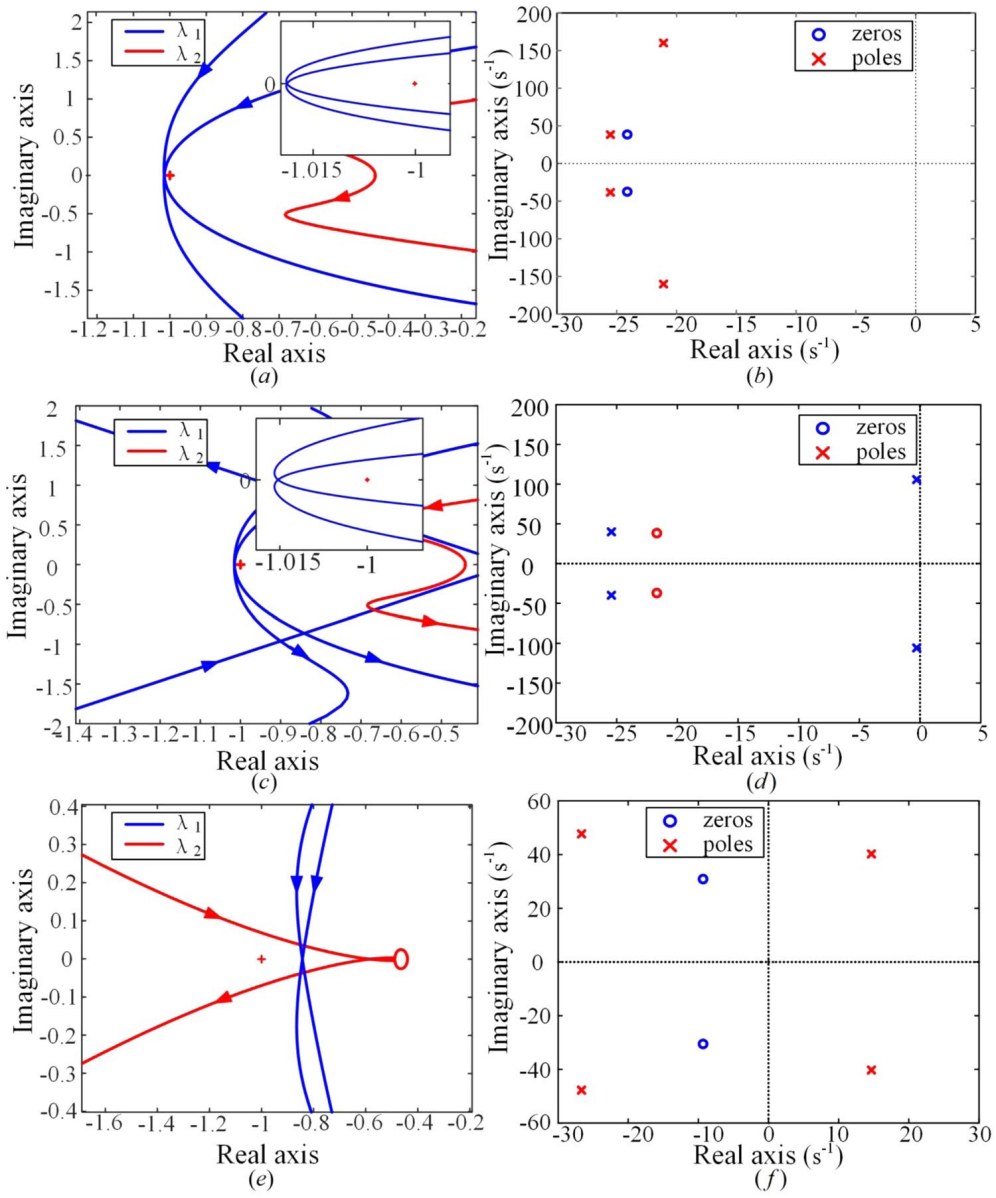
Comparative studies of small-signal frequency-domain stability analysis between the dynamic power flow theory and the standard modal analysis. On the left, the generalized Nyquist criterion method for MIMO systems is used, and on the right, the eigenvalue analysis is conducted by using the linear analysis tool in MATLAB. From top to bottom, different network strengths, *L*_*g*_ = *0.5*, *0.85*, and *1.5*, correspond to a stiff, critical, and weak grid, respectively.

The analytical results with the variation of grid strengths are given in the three left panels of Fig. 6. From top to bottom, *L*_*g*_ = *0.5*, *0.85*, and *1.5*, corresponding to a stiff, critical, and weak grid, respectively. In contrast, the right three panels of Fig. 6 are for the modal analysis of the same system, compared with the left ones by using the linear analysis tool in MATLAB. The modal analysis has been widely used and is reliable in determining small-signal stability of power systems. It is convincing to test our theory by comparing its results with modal analysis results^3^. Because the open-loop transfer function matrix has two Smith–McMillan poles on the right-half plane, the system is stable if there are two counter-clockwise encirclements of the characteristic loci around the (−1,0) point. This is exactly what we see in Fig. 6(a). The dominant poles in Fig. 6(b) are on the left side of the imaginary axis. Thus, the system is stable. Compared to Fig. 6(a), the characteristic loci in Fig. 6(c) are closer to the (−1,0) point, and in this situation we find that if we increase the inductance of the transmission line *L*_*g*_ a little bit, we will have a completely different pattern of plots. Thus, the system is regarded as critically stable. The dominant poles in Fig. 6(d) are nearly on the imaginary axis. However, if we change the grid parameter a little bit further, we will find that the dominant poles come across the imaginary axis. For a much larger *L*_*g*_, e.g., *L*_*g*_ = *1.5*, we see that in Fig. 6(e) the characteristic loci do not encircle the (−1, 0) point again, indicative of instability of the system, and in Fig. 6(f) the dominant poles are on the right half plane. Based on these comparisons, we find that the system stability and its change with grid strength derived from the generalized Nyquist criterion of MIMO systems are the same as those derived from the modal analysis, which has verified the validity of the small-signal linearized relation of the dynamic power flow given in the frequency domain.

From all these comparisons, we can see that our theoretical analysis results are consistent with the numerical simulation results including time-domain and frequency-domain results under different situations, and thus the mathematical relations are correct without any ambiguity.

## Discussion and Conclusions

In conclusion, for the first time we have developed a novel theory of dynamic power flow and found an explicit dynamic relation between the instantaneous (active and reactive) powers and voltage vectors. Based on this relation, the dynamic network characteristics can be well described. This relation can also be specified to the usual stationary power flow, the same as the classical power flow equations in the traditional power system analysis. Our theoretic results have been well verified by numerical simulations on transient analysis of a small power-electronics-based power system consisting of three VSCs connected to an infinite strong bus, and a small-signal frequency-domain stability analysis of a voltage source converter connected to an infinitely strong bus as well. Thus, these results are expected to be general and applicable to not only the traditional power systems, but also new-generation power-electronics-dominant power systems, and invaluable for a dynamic analysis of future power systems.

Finally, it is worthwhile to give some relevant statements as follows.

1. As the major role of a transmission network playing in a power system is to transfer power between different devices, which can be simplified as voltage sources based on the Thevenin equivalence in the circuit theory. Thus, both the dynamic external characteristics of devices and networks are of great importance in power system analysis. The novel dynamic power-flow relations for unveiling the dynamic characteristics of networks given in this paper are significant. We agree that only after understanding the complicated dynamic characteristics of network, can we solve the puzzles in the multi-time scale dynamical behaviors of power-electronics-dominant power systems^1^.
2. As is well known, the stationary power flow in the traditional power system has been widely used and is essentially important. However, the stationary power flow has shown its shortage in not reflecting relatively faster dynamic process of the system, as shown in Fig. 4. Since the proportion of power electronics devices in power systems is becoming higher gradually, the faster dynamic process of systems, including not only devices, but also grid, should be carefully examined. We expect that the dynamic power flow relation could play a similar active role in future power grids. One possible test case is the sub-synchronous oscillation^41^. In the traditional power system, even for the low-frequency oscillation analysis, as the oscillation frequency is much lower than the working frequency, the network dynamics can be ignored. However, in the sub-synchronous oscillation, as the frequency is around a dozen of Hz, comparable to and below the working frequency, the network dynamics was usually studied with various electromagnetic transient simulation software^42^.
3. For the short transmission line, usually a constant conductance (or a reactance working on the working frequency) is satisfying for the system description. However, for a medium-length transmission line, a nominal-π circuit should be used. In addition, for transmission lines in low-voltage (or medium-voltage) distribution networks, the resistance cannot be neglected compared to the reactance. Further work on the impact of capacitive elements on transmission lines within the same framework should be performed by including the dynamics of capacitors, and accordingly, the equation forms are expected to become more complicated.
4. The major objective of this paper is to provide a theoretical picture for the relation of the imbalanced powers and voltage magnitude and phase of the dynamical network, and to work as an interface in the amplitude-phase motion theory^31–35^. It would not substitute all existing electromagnetic transient simulations for calculating the instantaneous relation of the current and voltage on the network.
5. Finally, the concept of dynamic power flow has also been proposed by some other researchers in several recent works on integration of renewable energies, such as the photovoltaic system, wind power, etc.^43,44^. Nevertheless, they actually considered it in a much slower time scale, such as the maximum power point tracking due to the fluctuation of renewables, and were fundamentally different from ours. In addition, with the same objective to study the frequency variation effect of dynamic network characteristics of power-electronics-based power systems, similar equations for the instantaneous relation of voltages and currents have been analyzed and obtained in ref. ^45^. But they were further handled with an integral by part and finally described in a form of infinite series, whose convergence was not proved. In contrast, our results including differential and algebraic equations in this paper are rigorous.

## Appendix

Here we present the detailed derivation of the transfer matrix of the VSC in the AC current control time scale. The current loop PI control is given in (1). As the assumption in^35^, the dynamic of the voltage outer loop is neglected in the AC current control time scale, which regards the current reference value as constant. As the input of the current loop PI control is the imbalanced current in the PLL frame, we need to build the relationship between the current in the PLL frame and the power of the internal voltage as shown in (2) and (3). The subscript sands for the values in the internal voltage frame, whose D axis aligns with the internal voltage vector

1 $$[\begin{array}{c}\Delta {e}_{d}^{p}\\ \Delta {e}_{q}^{p}\end{array}]=[\begin{array}{cc}{k}_{p1}+{k}_{i1}/s & 0\\ 0 & {k}_{p1}+{k}_{i1}/s\end{array}][\begin{array}{c}-\Delta {i}_{d}^{p}\\ -\Delta {i}_{q}^{p}\end{array}]$$

2 $$[\begin{array}{c}\Delta {i}_{d}^{e}\\ \Delta {i}_{q}^{e}\end{array}]=\left[\begin{array}{cc}\frac{1}{{E}_{0}} & 0\\ 0 & \frac{1}{{E}_{0}}\end{array}\right][\begin{array}{c}\Delta P\\ \Delta Q\end{array}]+\left[\begin{array}{c}-\frac{{P}_{0}}{{E}_{0}^{2}}\Delta E\\ -\frac{{Q}_{0}}{{E}_{0}^{2}}\Delta E\end{array}\right]$$

3 $$[\begin{array}{c}\Delta {i}_{d}^{p}\\ \Delta {i}_{q}^{p}\end{array}]=[\begin{array}{cc}\cos ({\theta }_{p0}-{\theta }_{0}) & -\,\sin ({\theta }_{p0}-{\theta }_{0})\\ \sin ({\theta }_{p0}-{\theta }_{0}) & \cos ({\theta }_{p0}-{\theta }_{0})\end{array}]\,[\begin{array}{c}\Delta {i}_{d}^{e}\\ \Delta {i}_{q}^{e}\end{array}]+[\begin{array}{cc}-{i}_{q0}^{e}\,\cos ({\theta }_{p0}-{\theta }_{0}) & -{i}_{d0}^{e}\,\sin ({\theta }_{p0}-{\theta }_{0})\\ -{i}_{q0}^{e}\,\sin ({\theta }_{p0}-{\theta }_{0}) & {i}_{d0}^{e}\,\cos ({\theta }_{p0}-{\theta }_{0})\end{array}]([\begin{array}{c}\Delta {\theta }_{p}\\ \Delta {\theta }_{p}\end{array}]-[\begin{array}{c}\Delta {\theta }_{E}\\ \Delta {\theta }_{E}\end{array}])$$

The output of the current loop PI control is the internal voltage in the PLL frame. However, the output what we need is its amplitude and angle, so the following relationship is necessary

4 $$[\begin{array}{c}\Delta {\theta }_{E}\\ \Delta E\end{array}]=\left[\begin{array}{cc}-\frac{{e}_{q0}^{p}}{{E}_{0}^{2}} & \frac{{e}_{d0}^{p}}{{E}_{0}^{2}}\\ \frac{{e}_{d0}^{p}}{{E}_{0}} & \frac{{e}_{q0}^{p}}{{E}_{0}}\end{array}\right][\begin{array}{c}\Delta {e}_{d}^{p}\\ \Delta {e}_{d}^{p}\end{array}]+[\begin{array}{c}\Delta {\theta }_{p}\\ 0\end{array}]$$

The dynamic of PLL is considered, i.e.,

5 $$\Delta {\theta }_{p}=(\Delta {\theta }_{t}-\Delta {\theta }_{p})({k}_{p2}/s+{k}_{i2}/{s}^{2})$$

To eliminate variables of PLL and the terminal voltage, we need the following relations

6 $$[\begin{array}{c}\Delta {\theta }_{i}\\ \Delta I\end{array}]=\left[\begin{array}{cc}-\frac{{i}_{q0}^{p}}{{E}_{0}^{2}} & \frac{{i}_{d0}^{p}}{{E}_{0}^{2}}\\ \frac{{i}_{d0}^{p}}{{E}_{0}} & \frac{{i}_{q0}^{p}}{{E}_{0}}\end{array}\right][\begin{array}{c}\Delta {i}_{d}^{p}\\ \Delta {i}_{d}^{p}\end{array}]+[\begin{array}{c}\Delta {\theta }_{p}\\ 0\end{array}]$$

7 $$[\begin{array}{c}\Delta I\\ {I}_{0}\Delta {\theta }_{i}\end{array}]=\frac{1}{({s}^{2}+{\omega }_{0}^{2}){L}_{f}}[\begin{array}{cc}s & {\omega }_{0}\\ -{\omega }_{0} & s\end{array}]([\begin{array}{cc}\cos ({\theta }_{E0}-{\theta }_{i0}) & -\,\sin ({\theta }_{E0}-{\theta }_{i0})\\ \sin ({\theta }_{E0}-{\theta }_{i0}) & \cos ({\theta }_{E0}-{\theta }_{i0})\end{array}][\begin{array}{c}\Delta E\\ {E}_{0}\Delta {\theta }_{E}\end{array}]-[\begin{array}{cc}\cos ({\theta }_{t0}-{\theta }_{i0}) & -\,\sin ({\theta }_{t0}-{\theta }_{i0})\\ \sin ({\theta }_{t0}-{\theta }_{i0}) & \cos ({\theta }_{t0}-{\theta }_{i0})\end{array}][\begin{array}{c}\Delta {U}_{t}\\ {U}_{0}\Delta {\theta }_{t}\end{array}])$$

Then the initial values calculated in (8)–(14), where all the variables with d or q as subscript stand for the values in the terminal voltage coordinate system and x or y stands for those in the polar coordinate system rotating at the synchronous speed

8 $${\theta }_{t0}=\arcsin \left(\frac{{P}_{0}{X}_{g}}{{U}_{t0}{U}_{g}}\right)$$

9 $$\{\begin{array}{c}{i}_{d0}=\frac{{P}_{0}}{{U}_{t0}}\\ {i}_{q0}=\frac{{i}_{d0}\,\cos \,{\theta }_{t0}-{P}_{0}}{\sin \,{\theta }_{t0}}\end{array}$$

10 $$[\begin{array}{c}{i}_{x0}\\ {i}_{y0}\end{array}]=[\begin{array}{cc}\cos \,{\theta }_{t0} & -\,\sin \,{\theta }_{t0}\\ \sin \,{\theta }_{t0} & \cos \,{\theta }_{t0}\end{array}][\begin{array}{c}{i}_{d0}\\ {i}_{q0}\end{array}]$$

11 $${I}_{0}=\sqrt{{i}_{x0}^{2}+{i}_{y0}^{2}}$$

12 $$\{\begin{array}{c}{E}_{y0}=\frac{{P}_{0}({X}_{g}+{X}_{f})}{{U}_{g}}\\ {E}_{x0}=\frac{{P}_{0}-{i}_{y0}{E}_{y0}}{{i}_{x0}}\end{array}$$

13 $$\{\begin{array}{c}E=\sqrt{{E}_{x0}^{2}+{E}_{y0}^{2}}\\ {\theta }_{E0}=\arcsin \frac{{E}_{y0}}{{E}_{x0}}\end{array}$$

14 $${Q}_{0}={E}_{y0}{i}_{x0}-{E}_{x0}{i}_{y0}$$

After eliminating unnecessary variables using (1–7), we can obtain the transfer matrix of the VSC in the current control time scale by taking internal voltage as inputs and the powers on it as outputs. Such a process is presented in (15–18) by matrix operations

15 $$T=[\begin{array}{cccc}{T}_{11} & {T}_{12} & {T}_{13} & {T}_{14}\\ {T}_{21} & {T}_{22} & {T}_{23} & {T}_{24}\\ {T}_{31} & {T}_{32} & {T}_{33} & {T}_{34}\\ {T}_{41} & {T}_{42} & {T}_{43} & {T}_{44}\end{array}]$$

16 $$U=[\begin{array}{c}{U}_{11}\\ {U}_{21}\end{array}]$$

17 $$V=[\begin{array}{ccc}{V}_{11} & {V}_{12} & {V}_{13}\end{array}]$$

18 $${G}_{VSC}=V\cdot {T}^{-1}\cdot U$$

where

$$\begin{array}{c}{T}_{11}=[\begin{array}{cccc}1 & 0 & 0 & 0\\ 0 & -1 & 0 & 0\\ 1 & 0 & 0 & 0\\ 0 & 1 & 0 & 0\end{array}]{T}_{22}=[\begin{array}{cccc}1 & 0 & 0 & 0\\ 0 & 1 & 0 & 0\\ 0 & 0 & 1 & 0\\ 0 & 0 & 0 & 1\end{array}]{T}_{23}=[\begin{array}{c}-1\\ 0\\ -1\\ 0\end{array}]\\ {T}_{12}=[\begin{array}{cccc}0 & {P}_{0}/{E}_{0}^{2} & 0 & 0\\ 0 & {Q}_{0}/{E}_{0}^{2} & 0 & 0\\ {I}_{0}\,\sin ({\theta }_{E0}-\arctan ({i}_{y0}/{i}_{x0})) & 0 & -{I}_{0}\,\sin ({\theta }_{E0}-\arctan ({i}_{y0}/{i}_{x0})) & 0\\ {I}_{0}\,\cos ({\theta }_{E0}-\arctan ({i}_{y0}/{i}_{x0})) & 0 & -{I}_{0}\,\cos ({\theta }_{E0}-\arctan ({i}_{y0}/{i}_{x0})) & 0\end{array}]\end{array}$$

$$\begin{array}{c}{T}_{13}=[\begin{array}{c}0\\ 0\\ -\,\cos ({\theta }_{E0}-\arctan ({i}_{y0}/{i}_{x0}))\\ \sin ({\theta }_{E0}-\arctan ({i}_{y0}/{i}_{x0}))\end{array}]\\ {T}_{21}=[\begin{array}{cccc}0 & 0 & 0 & 0\\ 0 & 0 & 0 & 0\\ 0 & 0 & -\,\sin ({\theta }_{t0}-\arctan ({i}_{y0}/{i}_{x0}))/{I}_{0} & -\,\cos ({\theta }_{t0}-\arctan ({i}_{y0}/{i}_{x0}))/{I}_{0}\\ 0 & 0 & -\,\cos ({\theta }_{t0}-\arctan ({i}_{y0}/{i}_{x0})) & \sin ({\theta }_{t0}-\arctan ({i}_{y0}/{i}_{x0}))\end{array}]\\ {T}_{24}=[\begin{array}{cccc}-\,\sin ({\theta }_{t0}-{\theta }_{E0})/{E}_{0} & -\,\cos ({\theta }_{t0}-{\theta }_{E0})/{E}_{0} & 0 & 0\\ -\,\cos ({\theta }_{t0}-{\theta }_{E0}) & \sin ({\theta }_{t0}-{\theta }_{E0}) & 0 & 0\\ 0 & 0 & 0 & 0\\ 0 & 0 & 0 & 0\end{array}]\end{array}$$

$${T}_{33}=[1+{G}_{2}]$$

$${T}_{34}=[\begin{array}{cccc}0 & 0 & 0 & -{G}_{2}\end{array}]$$

$$\begin{array}{c}{T}_{42}=\left[\begin{array}{cccc}0 & 0 & 0 & 0\\ 0 & 0 & 0 & 0\\ {E}_{0}{G}_{3}(\frac{\sin \left({\theta }_{E0}-ac\,\tan \left(\frac{{i}_{y0}}{{i}_{x0}}\right)\right)\cdot s}{{\omega }_{b}}-{\omega }_{0}\,\cos \left({\theta }_{E0}-ac\,\tan \left(\frac{{i}_{y0}}{{i}_{x0}}\right)\right) & {G}_{3}(\frac{-\,\cos ({\theta }_{E0}-ac\,\tan \left(\frac{{i}_{y0}}{{i}_{x0}}\right)\cdot s}{{\omega }_{b}}-{\omega }_{0}\,\sin \left({\theta }_{E0}-ac\,\tan \left(\frac{{i}_{y0}}{{i}_{x0}}\right)\right) & 0 & 1\\ {E}_{0}{G}_{3}(\frac{-\,\cos \left({\theta }_{E0}-ac\,\tan \left(\frac{{i}_{y0}}{{i}_{x0}}\right)\right)\cdot s}{{\omega }_{b}}-{\omega }_{0}\,\sin \left({\theta }_{E0}-ac\,\tan \left(\frac{{i}_{y0}}{{i}_{x0}}\right)\right) & {G}_{3}(\frac{\cos ({\theta }_{E0}-ac\,\tan \left(\frac{{i}_{y0}}{{i}_{x0}}\right)\cdot s}{{\omega }_{b}}-{\omega }_{0}\,\sin \left({\theta }_{E0}-ac\,\tan \left(\frac{{i}_{y0}}{{i}_{x0}}\right)\right) & {I}_{0} & 0\end{array}\right]\\ {T}_{44}=\left[\begin{array}{cccc}1 & 0 & 0 & 0\\ 0 & 1 & 0 & 0\\ 0 & 0 & {G}_{3}\left({\omega }_{0}\,\sin \left({\theta }_{t0}-\arctan \left(\frac{{i}_{y0}}{{i}_{x0}}\right)\right)+\frac{\cos ({\theta }_{t0}-\arctan \left(\frac{{i}_{y0}}{{i}_{x0}}\right)s}{{\omega }_{b}}\right) & {U}_{t0}{G}_{3}(\frac{-\,\sin ({\theta }_{t0}-\arctan \left(\frac{{i}_{y0}}{{i}_{x0}}\right)s}{{\omega }_{b}}+{\omega }_{0}cos({\theta }_{t0}-\arctan \left(\frac{{i}_{y0}}{{i}_{x0}}\right)\\ 0 & 0 & {G}_{3}\left({\omega }_{0}\,\sin \left({\theta }_{t0}-\arctan \left(\frac{{i}_{y0}}{{i}_{x0}}\right)\right)-\frac{\cos ({\theta }_{t0}-\arctan \left(\frac{{i}_{y0}}{{i}_{x0}}\right)s}{{\omega }_{b}}\right) & {U}_{t0}{G}_{3}(\frac{\cos ({\theta }_{t0}-\arctan \left(\frac{{i}_{y0}}{{i}_{x0}}\right)s}{{\omega }_{b}}+{\omega }_{0}sin({\theta }_{t0}-\arctan \left(\frac{{i}_{y0}}{{i}_{x0}}\right)\end{array}\right]\end{array}$$

$${T}_{41}=[\begin{array}{cccc}0 & 0 & {G}_{1} & 0\\ 0 & 0 & 0 & {G}_{1}\\ 0 & 0 & 0 & 0\\ 0 & 0 & 0 & 0\end{array}]{T}_{43}={[0]}_{4\times 1}{T}_{14}={[0]}_{4\times 4}{T}_{31}={T}_{32}={[0]}_{1\times 4}$$

$${G}_{1}={K}_{p1}+\frac{{K}_{i1}}{s}{G}_{2}={K}_{p2}+\frac{{K}_{i2}}{s}{G}_{3}=\frac{{\omega }_{0}}{\left[{\left(\frac{s}{{\omega }_{b}}\right)}^{2}+{\omega }_{0}^{2}\right]\cdot {L}_{f}}$$

$${U}_{11}=\left[\begin{array}{cc}\frac{1}{{E}_{0}} & 0\\ 0 & \frac{1}{{E}_{0}}\end{array}\right]{V}_{12}=[\begin{array}{cc}1 & 0\\ 0 & 1\end{array}]{U}_{21}={[0]}_{11\times 2}{V}_{13}={[0]}_{2\times 7}{V}_{11}={[0]}_{2\times 4}$$
